# Occurrence of Virulence Genes and Antimicrobial Resistance of *E. coli* O157:H7 Isolated from the Beef Carcass of Bahir Dar City, Ethiopia

**DOI:** 10.1155/2021/8046680

**Published:** 2021-09-17

**Authors:** Habtamu Yalew Ayenew, Birhan Agmas Mitiku, Tesfaye Sisay Tesema

**Affiliations:** ^1^Department of Veterinary Science, College of Agriculture and Environmental Science, Bahir Dar University, Bahir Dar, Ethiopia; ^2^Institute of Biotechnology, Addis Ababa University, Addis Ababa, Ethiopia

## Abstract

*E*. *coli* O157:H7 is one of the most virulent foodborne pathogens. The aim of this study was to isolate *E. coli* O157:H7, determine virulence genes carried by the organism, and assess the antimicrobial susceptibility pattern of the isolates from beef carcass samples at Bahir Dar city. Swab samples (*n* = 280) were collected from the carcass of cattle slaughtered at the abattoir and processed using sorbitol MacConkey agar supplemented with cefixime telluride and confirmed with latex agglutination test. A polymerase chain reaction was performed on isolates for the detection of virulence genes *stx1*, *stx2*, *hlyA*, and *eae*. Antimicrobial susceptibility testing was performed using the disk diffusion method. Of 280 samples processed, 25 (8.9%) isolates were positive. Out of 25 isolates subjected for molecular detection, 8 (32%) and 14 (56%) isolates possessed *stx1* and *stx2* genes, respectively; from those, 5 (20%) isolates had both genes for the production of Shiga toxins. Compared from other virulent genes relatively higher proportion of 18 (72%) isolates carried the *hlyA* gene. Only 5 (2%) isolates were positive for *eae*. Resistance was detected in all 25 (100%) isolates and 3 (12%) against clindamycin and trimethoprim, respectively. This study result highlights the potential threat to public health. The abattoir workers need to be aware about the pathogen and should follow appropriate practices to prevent contamination of meat intended for human consumption.

## 1. Background

Foodborne pathogens are one of the leading causes of illness and death worldwide. They exert heavy burden costing billions of dollars in medical care, social costs, and overall economic and infrastructure effects of countries [1–3]. Developing countries including Ethiopia are more vulnerable to foodborne illnesses mainly due to the lack of awareness regarding safe and hygienic food practices [4]. About 2 million people die per year due to diseases of foodborne pathogens in developing countries including Ethiopia [2, 4].

Among the pathogenic *E*. *coli* strains, enterohemorrhagic *E*. *coli* O157:H7 is one of the most virulent foodborne strains which is the leading cause of hemorrhagic colitis (HC), hemolytic uremic syndrome (HUS), and thrombotic thrombocytopenic purpura (TTP) in human beings. These illnesses may lead to death due to improper absorption of nutrients and destruction of certain tissues in the target organs [5, 6]. The ability to produce one or more Shiga toxins is a hallmark of *E*. *coli* O157:H7 infection [7].

Shiga toxins are a family of related toxins with two major groups, Shiga toxin 1 (*stx1*) and Shiga toxin 2 (*stx2*), expressed by genes considered to be horizontally acquired by bacteriophages, and are encoded by *stx1* and *stx2* genes, respectively [5, 8]. In addition, enterohemolysin [9] and intimin [10], encoded by *hlyA* and *eae* genes, respectively, are another form of virulence factors of the bacteria [7].

It is estimated that Shiga toxin-producing *E. coli* (STEC) causes 2,801,000 acute illnesses each year worldwide and may cause 3,890 cases of HUS and 270 cases of end-stage renal disease [3, 11].

Cattle are the primary reservoirs of *E*. *coli* O157:H7, and ground beef and beef products are identified as major sources of foodborne transmission. Carcass contamination occurs through skin to carcass or fecal to carcass transfer of the pathogen during the slaughter process at processing plants [12, 13]. The process of removing the gastrointestinal tract during slaughtering of food animals is regarded as one of the most important sources of carcass and organ contamination with bacteria at abattoirs [14]. In addition to being gastrointestinal contamination sources, lymph nodes left at carcass also serve as niches of *E*. *coli* O157:H7 persistence, thus sources of carcass contamination [15].

The clinical significance and economic burden associated with outbreaks caused by *E*. *coli* O157:H7 have led to the development of a variety of detection methods. These include the application of conventional bacteriological methods using selective media such as sorbitol MacConkey agar or chromogenic agar, which usually take several days to complete, and molecular-based assays such as polymerase chain reaction- (PCR-) based methods, microarray, and whole genomic sequencing. Of these molecular methods, PCR is a commonly used method [16].

The use of antimicrobial agents in the treatment of *E. coli* O157:H7 infection is not recommended but remains a debatable issue. This is based on studies that have shown it to be a risk factor for the development of HUS [7]. Besides, the emergence of antibiotic-resistant bacteria and their resistance genes has turned into a serious growing issue in current medications [17].

In Ethiopia, foodborne disease is a common problem. This is due to the prevailing poor food handling and sanitation practices, inadequate food safety laws, weak regulatory systems, lack of financial resources to invest in safer equipment, and lack of education for food handlers [4, 18]. In addition to that animals are commonly slaughtered and dressed under unhygienic conditions, this further compromises the microbiological quality and safety of meat obtained from the animals. The magnitude of contamination may vary from area to area based on the management of slaughtering practice [19, 20]. However, only very few studies can be found regarding *E*. *coli* O157:H7 in animals, animal products, or people in Ethiopia [13, 20, 21].

As far as our knowledge is concerned, few data were available in Bahir Dar regarding carcass contamination of *E*. *coli* O157:H7 during the slaughtering process. The lack of vigorous surveillance of the pathogen in Ethiopia meat and meat products presents a challenge for risk-based approaches to improve food safety as it becomes difficult to demonstrate the magnitude of contamination with this pathogen during the slaughtering process. There is a need to generate more data regarding *E. coli* O157:H7 from abattoirs by taking samples from the sites of the carcass which are more prone to contamination (i.e., the abdomen (flank), thorax (lateral), and breast (lateral)) during the slaughtering process.

The current accepted standard procedure for *E. coli* O157:H7 identification is amplification of *stx1*, *stx2*, *eae,* and *hlyA* by PCR [21, 22]. PCR has gained more acceptance and use due to its ability to differentiate and reliability. In addition, various target genes have been used in the PCR detection scheme for the pathogen [16]. However, very few studies can be found regarding identification of the bacteria based on the detection of virulence genes in Ethiopia [6], and little work was done in the Bahir Dar municipal abattoir or any slaughtering areas at Bahir Dar and its environs.

The misuse of antimicrobial agents for farming and therapeutic purposes in animals and humans is the main cause of the emergence and transmission of antibiotic-resistant strains, which are very difficult to treat with commonly used antibiotics, to humans via the food supply chain [9]. Antimicrobial resistance among enteric bacteria is an increasing global public health concern. The widespread administration of antimicrobials promotes the selection of antimicrobial-resistant strains, which complicates the treatment of bacterial infections [23]. To our knowledge, there are few reports on the antibiotic resistance status of *E*. *coli* O157:H7 isolated from the abattoir in Ethiopia, and few studies have been carried out in Bahir Dar. Therefore, surveillance of antimicrobial resistance in *E*. *coli* O157:H7 is very important for preventing the spread of antimicrobial resistance in organisms and future disease management.

The aim of this study was to investigate the presence of the *E*. *coli* O157 strain in the carcass of cattle slaughtered at the Bahir Dar municipal abattoir, examine the presence of *stx1*, *stx2*, *eae,* and *hlyA* genes, evaluate the antimicrobial resistance of the isolates, and assess knowledge, attitude, and practice of abattoir workers towards food safety.

## 2. Methods

### 2.1. Description of the Study Area

**Figure 1:**
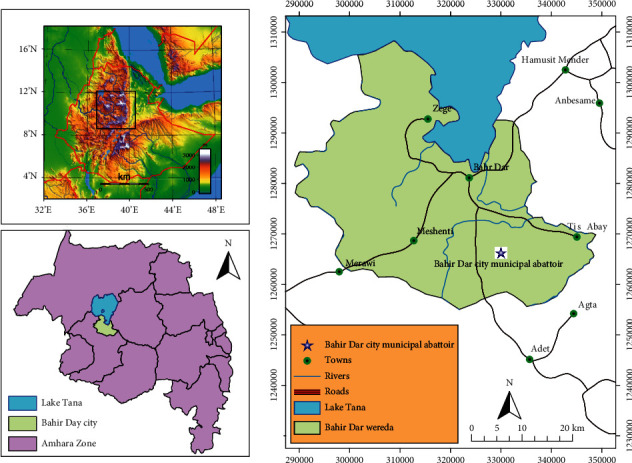
A map indicating the location of the study area.

The study was conducted at Bahir Dar city. Bahir Dar city is in the northwestern part of Ethiopia, 565 km away from Addis Ababa at latitude 11°35′37.10″ N and longitude 37°23′26.77″E on the south of Lake Tana, the upper water shed of the Blue Nile River (Figure 1) [24]. Cattle slaughtered at the Bahir Dar city abattoir are mainly of the zebu type and originated from different districts of northwest Ethiopia [25].

### 2.2. Study Design and Sampling Techniques

A cross-sectional study design was conducted from December 2019 to August 2020. Systematic random sampling at the abattoir was carried out to select the beef carcass. A swab was taken from the area of the carcass which was exposed for contamination during the slaughtering process. A total of 280 samples were taken to increase the probability of acquiring many isolates of the bacteria from the carcass.

The selected carcasses were swabbed using the method described in [26]. Sterile cotton-tipped swab (2 × 3 cm), fitted with shaft soaked with approximately 10 ml of buffered peptone water, was rubbed horizontally first and then vertically several times on the carcasses. The abdomen (flank), thorax (lateral), and breast (lateral) were sites selected for the occurrence of contamination and thus for sampling using swabbing [26]. All the swab samples were put inside the test tube. The samples for the study were collected under strict aseptic procedures and then were transported in an ice box to the Amhara Public Health Institute Microbiology Laboratory for further processing. Upon arrival, all the samples were stored at 4°C until to be processed for isolation and identification. The molecular detection and antimicrobial susceptibility determination were conducted at the Institute of Biotechnology, Addis Ababa University.

### 2.3. Isolation of *E. coli* O157:H7

The method was based on enrichment in a selective broth medium (modified Tryptone Soya Broth (TSB) supplemented with novobiocin (Oxoid) (10 mg/l)). The nonselective pre-enrichment technique was used to effectively recover low levels of stressed *E. coli* O157:H7. After that, the enrichment broths were prewarmed to prevent cold shocking of the organisms and to slow their initial growth [11, 27]. Then, plating on 1% sorbitol MacConkey agar (supplemented with tellurite-cefixime supplement (HiMedia) (CT-SMAC)) was incubated at 37°C for 24 hours. Nonsorbitol-fermenting (NSF) *E. coli* (colorless or pale colonies) were considered as presumptive *E*. *coli* O157:H7, whereas pinkish colored colonies (sorbitol fermenters) were considered as non-O157:H7 *E. coli* since *E. coli* O157:H7 does ferment sorbitol slowly [28].

### 2.4. Serotyping

All NSF colonies which were presumptive *E*. *coli* O157:H7 were subjected to slide agglutination with the *E*. *coli* O157 latex test kit according to the manufacturer's instructions. Isolates giving a positive reaction (formed agglutination) to the latex test were considered to be *E*. *coli* O157:H7 positive [29]. Once confirmation testing was completed, isolates were stored in a 20% glycerol TSB solution and frozen for further molecular characterization and conducting antimicrobial susceptibility analysis [30] and were transported and processed at the Institute of Biotechnology, Addis Ababa University.

### 2.5. Antibiotic Susceptibility Test

The antibacterial susceptibility testing of the isolates was performed using disc diffusion according to [31] using eleven antibiotic discs (all from Oxoid (oxytetracycline (30 *µ*g), tetracycline (30 *µ*g), chloramphenicol (30 *µ*g), ampicillin (10 *µ*g), sulphonamides (30 *µ*g), ciprofloxacin (5 *µ*g), neomycin (10 *µ*g), clindamycin (10 *µ*g), trimethoprim (5 *µ*g), norfloxacin (10 *µ*g), and streptomycin (25 *µ*g)). The selection criteria for discs depended on the regular use of antimicrobials in the ruminants and their potential public health importance [32].

The antibiotic discs were firmly placed on sterile Mueller–Hinton agar plates previously seeded with a 24-hour-old culture of the isolate (106 CFU/ml of 0.5 McFarland standard). The plates were incubated at 37°C for 24 hr, and diameters of zones of inhibition were compared. Multiple antibiotic-resistant isolates were defined as resistance to greater than or equal to three classes of the antibiotics tested. Plates were incubated at 37°C, and the diameter of the zone of growth inhibition was measured with calipers to the nearest millimeter and interpreted accordingly [22, 31].

### 2.6. Detection of Virulence Genes

Standard methods of the boiling method of DNA extraction were carried out [33, 34]. The sorbitol-negative isolates which were identified by latex agglutination test were examined by the polymerase chain reaction (PCR) (HiMedia Laboratories PLC.) to determine the presence of *stx1*, *stx2*, *eae*, and *hlyA* virulence genes [21, 22, 34–37]. PCR sequence of primers, their product size, and their amplification conditions are depicted in Table 1.

### 2.7. Ethical Review

The study protocol was reviewed and approved by the Institutional Review Board of Bahir Dar University, College of Agriculture and Environmental Science, School of Animal Production and Veterinary Medicine. A letter of support was obtained from Bahir Dar University, and official permission was requested from the concerned higher officials, Bahir Dar city Abattoir Administration.

### 2.8. Data Analysis

All data collected during the study period were checked, coded, and entered into a computer Excel spreadsheet (Microsoft Excel 2013) and analyzed by SPSS software, version 23. Descriptive statistics such as percentages and frequency distribution were applied to quantify magnitude of contamination of carcass, molecular detection levels, and antimicrobial susceptibility profile of the isolates. Pearson's chi-square was applied to measure the difference in the isolation of *E. coli* O157:H7 by the site of the sampled carcass. A *P* value <0.05 was considered indicative of a statistical significance.

## 3. Results

### 3.1. Isolation and Identification of *E. coli* O157:H7

A swab sample was taken from apparently healthy cattle slaughtered in the abattoir. Standard microbiological examination of the swab samples collected from different regions of the carcass (the abdomen (flank), thorax (lateral), and breast (lateral)) and serotyping of the isolated *E. coli* O157:H7 with latex agglutination revealed the isolation of 25 (8.9%) strains of *E. coli* O157:H7 organisms out of a total of 280 examined samples. Although swab samples from the thorax region yield the highest isolation rate of the organism, no significant difference (*P* > 0.05, 95% CI) occurred among the regions of the carcass where the sample was taken (Table 2).

### 3.2. Detection of Virulence Genes

Out of 25 isolates tested by PCR for the four virulence genes (*stx1*, *stx2*, *eae*, and *hlyA*), 1 (4%) isolate had all the four virulence genes. Four (16%) of the isolates had at least three of the genes in their DNA structure, and 25 (100%) of isolates had at least one of the four virulence genes (Table 3).

**Figure 2:**
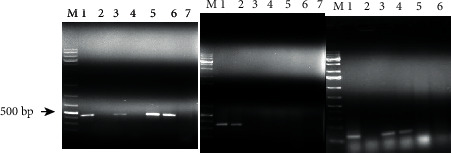
Agarose gel electrophoresis of amplified products for *eae* (490 bp), *stx2* (349 bp), and *stx1* (110 bp) of *E. coli* O157:H7 isolates, respectively. Key: lane M: DNA ladder, lane 1: known positive control (taken from previous works), lanes 2–6: samples (those which have a band are positive to the gene), and lane 7: negative control.

With regard to each gene, 8 (32%) isolates were positive for *stx1*, 14 (56%) of the isolates had the *stx2* gene, 5 (20%) isolates had both genes (i.e., *stx1* and *stx2*), 18 (72%) isolates had the *hlyA* gene, and only 5 (2%) isolates had the *eae* gene. Typical gel images showing the agarose-separated PCR products of the virulence genes are indicated in Figure 2.

### 3.3. Antimicrobial Susceptibility Profile of *E. coli* O157:H7 Isolates

Out of 11 antimicrobials tested for the resistance profile, all isolates were susceptible (100%) to oxytetracycline, tetracycline, sulphonamides, ampicillin, ciprofloxacin, neomycin, and norfloxacin (Table 4). Resistances to three or more drugs were not detected in any of the isolates. All 25 (100%) isolates were resistant to clindamycin, and 3 (12%) isolates were resistant to trimethoprim. In addition, 3 (12%) isolates showed intermediate susceptibility to streptomycin.

## 4. Discussion

### 4.1. Isolation and Identification of *E. coli* O157:H7

The frequency of isolation of *E. coli* O157:H7 in swab samples taken from the carcass in this study was 8.9%. This result was in agreement with other reports in different countries with 8% in Debre Zeyit and Modjo [21] and 8.1% in Modjo, Ethiopia [12], and 9.3% in Jimma [40], but these data indicated high frequency of occurrence relative to reports from Haramaya University (2.2%) and Dire Dawa slaughterhouses (4%) [2] and swab samples taken from the carcass in the Hawassa municipal abattoir (2.7%) [19], Modjo (4.17%) [20], China (2.7%) [41], and Egypt (3.1%) [42]. On the contrary, it was lower than the culture-based prevalence of *E. coli* isolates (22.2%) from meat samples collected from the Mekelle municipal abattoir in northern Ethiopia [43]. This varied prevalence in various studies might be due to ecological variations among the study sites and the difference in the slaughtering procedure in different sites [12, 40]. Any detection of *E. coli* O157:H7 in meat is considered unacceptable [44]. However, there are no regulations in Ethiopia to protect meat consumers from foodborne pathogens such as *E. coli* O157:H7 [23].

### 4.2. Detection of Virulence Genes

Several virulence factors have been associated with the pathogenicity of NSF *E*. *coli* O157:H7. These factors include production of at least one of two Shiga toxins (*stx1* and/or *stx2*), intimin (*eae*), and enterohemolysin (EHEC-*hlyA*) [22]. In the present study, we investigated the existence of these four virulence genes in the isolated *E. coli* O157:H7 strains. All (100%) of the isolates had at least one of the four virulence genes (*stx1*, *stx2*, *eae*, and *hlyA*) in their DNA. Although this report was not in agreement with Kalin et al. [35] in which no virulence genes were detected except the *eae* gene, it was in line with the report from Turkey [45] in which all the isolates harbored the four virulence genes.

The pathogenicity of *E. coli* O157:H7 is attributed to the production of Shiga toxins (*stx1* and *stx2*), previously known as verocytotoxin because of their toxicity on Vero cells [46, 47]. PCR verified the presence of the *stx2* gene at the expected molecular size of 350 bp in 56% isolates which were more than *stx1* (only 32% isolates harbored it). This finding was in agreement with studies in California [47], China [41], Egypt [42], and Modjo, Ethiopia [20]. The strains carrying *stx2* are potentially more virulent than those carrying *stx1* or even strains carrying both *stx1* and *stx2* [22, 42, 47]. On the contrary, 36% of the isolates do not have either of the two Shiga toxin-producing genes. This result was in agreement with the research from Ohio, United States of America, with 23% *stx*-negative *E. coli* O157:H7 [48]. The role of *stx*-negative *E. coli* O157:H7 in the ecology and epidemiology of the human disease causing STEC is unknown. Although *stx* is considered to be the essential virulence factor of STEC, it has been suggested that *stx*-negative O157:H7 can cause diarrhea and HUS [48].

In the present study, 8% of the isolates have both *hlyA* and *eae* genes. The enterohemorrhagic hemolysin *hlyA* makes adherence possible for *eae*-negative strains. When both *hlyA* and *eae* genes are present, this is an indicator for increased pathogenicity [10]. In addition, 20% of the isolates harbored the *eae* gene in our study. The gene was mostly reported in STEC strains associated with severe human illnesses such as bloody diarrhea and HUS. However, STEC strains lacking the *eae* gene have been reported to cause outbreaks [42, 47]. In addition, *hlyA* is carried on the plasmid, and the bacteria can lose the gene together with the plasmid upon subsequent culturing, and this could be one reason for not detecting *hlyA* in all the isolates [49].

### 4.3. Antimicrobial Susceptibility Profiles of the Isolates

The *E. coli* O157:H7 strains isolated in this study were all found to be susceptible to most antimicrobials tested. Similar findings have been reported by other researchers [12, 19, 21]. However, resistant strains do exist mainly against clindamycin and trimethoprim. Clindamycin is effective against Gram-positive and anaerobic bacteria, but it is not effective against *E. coli* [50]. Antimicrobial resistance emerges from the use of antimicrobials in animals and humans and the subsequent transfer of resistance genes and bacteria among animals, humans, animal products, and the environment [51].

Intermediate resistance to streptomycin was also observed, while this result was opposite to a previous work conducted by Tilahun and Engdawork [52] from Hawassa who isolated *E. coli* O157:H7 from fish, and tolerance to streptomycin had not been detected in the study, but only moderate resistance was detected. Although streptomycin is one of the most commonly available drugs for use among livestock in Ethiopia, it is readily available in different dosage forms and in combination with other antimicrobials and vitamins [23].

Electiveness of treatments and ability to control infectious diseases in both animals and humans may be severely hampered. The recommended management of an infection mainly relies on supportive therapy and hydration [18]. Some of the drugs we tested such as ampicillin, sulfonamides, and trimethoprim can increase the risk of HUS; and thus, they are not recommended for the treatment of infections caused by *E. coli* O157:H7 [53].

Abattoir is one of the food industries that contributes to the problem of possible foodborne diseases and potential health hazards associated with food unless the principles of food hygiene are implemented [44, 54]. This study finding indicates the presence of microbiological contamination of indicator bacteria in beef meat in Bahir Dar city, and thus it is microbiologically unsafe for human consumption.

The limitation of this study was that it assessed only abattoir carcasses, excluding butcher shops and restaurants where contamination can occur at these stages. There may be backyard slaughter and cross-contamination in the process. The reason was due to the limited time frame and insufficient resources while conducting the research.

## 5. Conclusion and Recommendations

In general, from 280 swab samples taken from the carcass of cattle slaughtered at the Bahir Dar municipal abattoir, 25 (8.9%) samples were found contaminated with virulent *E. coli* O157:H7. All the isolated strains of *E. coli* O157:H7 were able to elicit at least one of the virulence genes (*stx1*, *stx2*, *eae*, and *hlyA*) subjected for PCR virulence gene detection which highlights the potential threat to public health both as a result of the contamination of the carcass and harboring of virulence genes. Thus, preventive approaches should be followed to control *E. coli* O157:H7 contamination in cattle slaughtering and processing chain by imposing strict hygienic meat processing practices in the Bahir Dar municipal abattoir. Intensive training should be given to those personnel working in municipal slaughterhouses to ensure the hygienic practices during slaughtering of animals. Improvement of facilities should be implemented with closure of substandard facilities and focusing resources on fewer facilities to improve meat hygiene in this resource-limited setting. Further detailed epidemiological and molecular studies should be carried out on *E. coli* O157:H7 in different abattoirs and species of food animals in the country. In this study, even if most of the isolates used are sensitive to the antimicrobials used, some isolates showed moderate resistance to trimethoprim and streptomycin. Thus, the status of antibiotic resistance in meat-borne pathogens such as *E. coli* O157:H7 should be monitored regularly.

## Figures and Tables

**Table 1:** Sequence of primers, their product size, and their annealing temperature.

Gene	Primer	Sequence (5′-3′)	Primer length (bp)	Annealing temperature (°C)	Reference
*stx1*	EVS-1	F: ATCAGTCGTCACTCACTGG	110	55	[38]
	EVC-2	R: CTGCTGTCACAGTGACAAA			
*stx2*	EVT-1	F: CAACACTGGATGATCTCAG	350	55	[38]
	EVT-2	R: CCCCCTCAACTGCTAATA			
hlyA	Hly-1	F: GGTGCAGCAGAAAAAGTTG	165	45	[39]
	Hly-1	R: CCACGTCGTCTTTTTCAACA			
*eae*	EAE-1	F: AAACAGGTGAAACTGTTGCC	490	55	[20]
	EAE-2	R: CTCTGCAGATTAACCTCTGC			

EAE: effacing and attaching; EV: verocytotoxin; *stx*: Shiga toxin; bp: base pair; F: forward primer; R: reverse primer; *hlyA*: hemolysin.

**Table 2:** Number of positive samples per region of the carcass sampled.

Carcass region	Number of samples taken	Number (%) of positive samples	*χ*^2^ value	*P* value
Abdomen	93	7 (28)	1.407	0.495
Thorax	94	11 (44)		
Breast	93	7 (28)		
Total	280	25 (100)		

**Table 3:** Virulence gene profile of the isolates.

Isolates	*stx1*	*stx2*	*eae*	*hlyA*
1	+	−	−	+
2	−	+	−	−
3	−	−	+	−
4	−	+	−	+
5	−	+	−	+
6	+	+	−	+
7	−	−	+	−
8	−	+	+	−
9	−	+	−	+
10	+	−	−	+
11	+	+	−	+
12	−	−	+	+
13	−	−	−	+
14	−	+	−	+
15	−	+	−	+
16	+	+	−	+
17	−	−	−	+
18	+	−	−	−
19	+	+	−	+
20	−	+	−	−
21	−	−	−	+
22	−	+	−	−
23	+	+	+	+
24	−	−	−	+
25	−	−	−	+
Total	8	14	5	18

**Table 4:** Antimicrobial susceptibility profile of *E. coli* O157:H7 isolated from carcass swab samples.

Drugs	Concentration (*µ*g)	Frequency of resistance and susceptible *E*. *coli* O157:H7 isolates		
		Resistant	Intermediate	Susceptible
Oxytetracycline	30	0 (0%)	0 (0%)	25 (100%)
Tetracycline	30	0 (0%)	0 (0%)	25 (100%)
Sulphonamides	30	0 (0%)	0 (0%)	25 (100%)
Ampicillin	10	0 (0%)	0 (0%)	25 (100%)
Ciprofloxacin	5	0 (0%)	0 (0%)	25 (100%)
Neomycin	10	0 (0%)	0 (0%)	25 (100%)
Clindamycin	10	25 (100%)	0 (0%)	0 (0%)
Trimethoprim	5	3 (12%)	0 (0%)	22 (88%)
Norfloxacin	10	0 (0%)	0 (0%)	25 (100%)
Streptomycin	25	0 (0%)	3 (12%)	22 (88%)
Chloramphenicol	10	0 (0%)	0 (0%)	25 (100%)

## Data Availability

The raw data used and/or analyzed during this study are available from the first author upon reasonable request.
